# Editorial: Synthesizing memory: integrating across fields and levels of scale

**DOI:** 10.3389/fcogn.2025.1637173

**Published:** 2025-06-17

**Authors:** Marian E. Berryhill, Lauren L. Richmond

**Affiliations:** ^1^Department of Psychology, University of Nevada, Reno, Reno, NV, United States; ^2^Department of Psychology, Stony Brook University, Stony Brook, NY, United States

**Keywords:** memory, working memory, memory research, theoretical models, synthesis

The goal of this Research Topic was to bring together researchers across various subfields of memory research to cast a new eye on operational definitions, methods, gaps in the field, and to promote synthesis. This follows efforts by others in a similar vein (Brainerd and Reyna, 2001; Festini and Reuter-Lorenz, 2013; Criss and Howard, 2015). We were inspired by the observation that memory subfields often operate in silos, which hampers theoretical resolutions and impedes growth. The Research Topics collected here provide novel perspectives and motivate future research that follows a more integrative approach to studying memory. Here, we summarize these articles to provide an overview of the Research Topic under two overarching themes: how memory is conceptualized and the intersection of attention, working memory, and long-term memory.

## Conceptualizing memory

Several articles provide persuasive arguments for expanding memory research to revisit the nature of memory and to address specific questions using particular methods. First, Finley revisits the foundation of memory by proposing expansions to the existing topology of memory. Finley defines memory as *information conveyed over time*, and he makes a strong case for expanding the scope of “memory” to include external memory, stored in the heads of others or accessed via technology. Modern humans' memory is now heavily supported by external cues including photos, reminders, and internet posts. He also advocates for an expanded definition of memory that includes “collective memory” that extends an individual's memory to communities' shared social memories including culture, customs, history across generations. Finally, Finley argues for inclusion of a novel conceptualization of “somatic memory.” He explains how past experiences reflect learning in the immune system, epigenetics, scars, and other bodily systems. This broad, provocative perspective encourages us to update definitions to reflect factors shaping memory in today's world.

An empirical submission by Sexton et al. investigated multiple aspects of “relational memory” in young and older adults. They rightly target the various ways the term relational memory is used and implement the *All Manner of Relations* (AMR) task to evaluate relational memory broadly, including item, spatial, associative, and sequential tasks. They found significant deficits in the older adult group across all forms of relational memory, with older adults exhibiting larger deficits in the sequential and spatial aspects of relational memory compared to associative memory. These data provide greater insight regarding the pattern of deficits across different aspects of relational memory, and encourage future research aimed toward characterizing performance across these various forms of relational memory.

Several papers identify a knowledge gap that could be addressed by a specific experimental approach. For example, Kalra reviews the use of Jacoby's *process dissociation procedure* (PDP). The PDP can reliably separate familiarity and recollection in episodic memory. The insight offered here is that the PDP could and should be used to disentangle other concurrent processes. The example put forward in the paper involves the use of a modified PDP to disentangle contributions from procedural memory *and* declarative memory in category learning. Future research implementing more ecologically valid tasks that make use of PDP are needed to disambiguate the contributions of multiple memory processes.

## Intersection of attention, working memory, and long-term memory systems

Phylactou et al. provide an overview of current theoretical debates in visual working memory (VWM) and demonstrate that advances in experimental techniques can drive theory by producing new insights. They make the point that current arguments are stymied by methodological limitations, and they recommend greater use of dual TMS designs engaging cortico-cortico paired associative stimulation to move theoretical debates forward. The temporal and spatial specificity of TMS permits causal testing of functional contributions to VWM and therefore offers a new way forward to leverage our current understanding and to test adjudicating predictions between representations of VWM. This aspirational approach looks forward to resolving theoretical disagreement as we advance our mechanistic understanding of various forms of memory.

A second persuasive review provides compelling evidence for a more expansive view of the medial temporal lobes (MTL) in memory and beyond. Hawkins and Yonelinas summarize the wealth of evidence showing MTL engagement during visual perception and visual working memory. As relational complexity of memoranda increases, there is greater reliance on the MTL, particularly the hippocampus. However, Hawkins and Yonelinas point out that there are interesting nuances; in addition to the classic link between MTL activity and episodic recollection, there is also a role for MTL activation in working memory specific to familiarity. The second part of the paper assembles supportive evidence from auditory perception, working memory, and long-term memory. Auditory forms of memory are much less studied, and this timely summary suggests that more work is needed to determine process general vs. modality specific processing requirements.

Popoviciu and Richmond make a broader argument for greater attention in the memory literature to errors of *commission*. These are memory errors associated with reporting or endorsing additional items outside of the original memory set. These errors complement *omission* errors (failures to report a previously studied item). Whereas disparate literatures have attended to commission errors, including research on false memory (e.g., using the Deese-Roediger-McDermott paradigm), associative memory, and hyperbinding, the broader memory literature has largely neglected commission errors. However, findings from these literatures provide unique insights into the role of age-related reductions in attentional capacity and control, inhibition, and increased gist-based processing in promoting such errors. Popoviciu and Richmond highlight the utility of characterizing both omission and commission errors to provide a richer understanding of age-related memory changes.

## Summary

Across these contributions, several common themes emerge that bring together disparate elements from traditionally siloed fields of memory research. Integration across topics is important as the field of memory research matures. These manuscripts offer tangible steps forward for the continued growth of the field that we hope will serve to guide future efforts in pursuit of this goal.
